# Whole-transcriptome sequence analysis of differentially expressed genes in *Phormium tenax* under drought stress

**DOI:** 10.1038/srep41700

**Published:** 2017-01-30

**Authors:** Zhen-yu Bai, Tong Wang, Yin-huan Wu, Ke Wang, Qian-yu Liang, Yuan-zhi Pan, Bei-bei Jiang, Lei Zhang, Guang-li Liu, Yin Jia, Qing-lin Liu

**Affiliations:** 1 Department of Ornamental Horticulture, Sichuan Agricultural University, 211 Huimin Road, Wenjiang District, Chengdu, Sichuan 611130, P.R. China

## Abstract

*Phormium tenax* is a kind of drought resistant garden plant with its rich and colorful leaves. To clarify the molecular mechanism of drought resistance in *Phormium tenax*, transcriptome was sequenced by the Illumina sequencing technology under normal and drought stress, respectively. A large number of contigs, transcripts and unigenes were obtained. Among them, only 30,814 unigenes were annotated by comparing with the protein databases. A total of 4,380 genes were differentially expressed, 2,698 of which were finally annotated under drought stress. Differentially expression analysis was also performed upon drought treatment. In KEGG pathway, the mechanism of drought resistance in *Phormium tenax* was explained from three aspects of metabolism and signaling of hormones, osmotic adjustment and reactive oxygen species metabolism. These results are helpful to understand the drought tolerance mechanism of *Phormium tenax* and will provide a precious genetic resource for drought-resistant vegetation breeding and research.

Drought is an important environmental threat, which affects the growth and crop yield of plants around the world. Previous studies about the model plant *Arabidopsis* have shown that plants respond to abiotic stresses by performing morphological and physiological characteristics changes^1^. Under drought conditions, many genes are involved, which even more lead to a range of physiological and biochemical alterations, changes in main biosynthetic pathways, photosynthesis, the antioxidation pathway and the respiration pathway, etc.^2^. There are two aspects for plant genes to cope with water shortage: those protecting plants resist environmental threat directly and those regulating down-stream target genes’ expression^3^. The first group mainly includes enzymes in various osmoprotectants’ biosynthesis, late-embryogenesis-abundant (LEA) proteins, antifreeze proteins, chaperones and detoxification enzymes. The second group includes regulatory proteins, such as transcription factors, protein phosphatases and protein kinases^3^.

*Phormium tenax* is a potential garden plant, because of its rich color and strong adaptability. Its roots are well developed and can grow under drought stress. Drought tolerance of *Phormium tenax* has attracted the attention of researchers^4^, but the molecular level has not yet carried out any research on its drought-resistant mechanism. It is currently high-throughput techniques have been widely used in the study of molecular mechanisms of plants in the stress response. The previous transcriptome sequencing analysis of plants under drought stress has been reported, such as poplar^5^, tomato^6^, cotton^7^. Because of its drought tolerance, *Phormium tenax* is an ideal species to study the adaptation of plants to drought stress. In this study, a *de novo* transcriptome sequencing that used the Illumina sequencing has been initiated in *Phormium tenax* and a functional genomics resource was obtained. A total of 4,063,419 contigs, 175,649 transcripts and 75,265 unigenes were identified. Further differential expression analysis, especially the Kyoto Encyclopedia of Genes and Genomes (KEGG) pathway analysis, not only help to understand the molecular basis of plant adaptation to drought environment from a new perspective, but also provide new ideas and excellent genetic resources for molecular breeding of water-saving and drought-resistant crops.

## Results

Study of the resistance of *Phormium tenax* to drought related to both the morphological indicators and physiological characteristics. Morphological indicators include length of taproot, lateral root length and the shoot height, while physiological characteristics include superoxide dismutase (SOD), peroxidase (POD), catalase (CAT), soluble protein, soluble sugar, malondialdehyde (MDA) and proline which should be determined under normal and drought conditions. The results showed that *Phormium tenax* under drought stress has longer taproot and lateral root compared to those grown in normal condition, while the shoot height was opposite. In addition, there has been a slight increase in the seven physiological characteristics during drought stress in *Phormium tenax* (Figure S1).

After data filtering, in total, 49,519,109 reads with 92.59% Q30 bases were selected. Using the *de novo* assembly program Trinity, short-reads were assembled into 4,018,976 contigs and 175,649 transcripts with a mean length of 54.92 bp (Figure S2A) and 856.06 bp (Figure S2B), respectively. The transcripts were subjected to cluster and assembly analyses. Totally 75,265 unigenes with a mean length of 565.88 bp were obtained, among which 21,864 genes (29.05%) have more than 500 bp in length (Figure S2C). The assembly results of contigs, transcripts and unigenes are revealed in Table S1. The N50 values of contigs, transcripts and unigenes were 49 bp, 1,396 bp and 919 bp, respectively.

All assembled unigene sequences were matched against the National center for Biotechnology (NCBI), nonredundant protein (Nr) database, Swiss-Prot, Pfam, Gene Ontology (GO), eukaryotic Orthologous Groups (KOG), KEGG and Cluster of Orthologous Groups of proteins (COG). Only 30,814 (40.9%) unigenes were annotated. According to the BLAST results, a total of 30,027 (39.9%), 20,891 (27.8%), 18,459 (24.5%), 16,809 (22.3%), 17,463 (23.2%) unigenes respectively were matched in the Nr, Swiss-Port, Pfam, GO and KOG database. The total functional annotation is listed in Table S2. Through the sequencing technology, only 30,814 (total 75,265) unigenes were matched to known genes, it may due to a shortage of sequences or the conserved functional domains of *Phormium tenax* in the public databases.

The result of BLAST search analysis showed that a total of 9,254 (31%), 7,683 (26%), 2,713 (9%) unigenes in the NR annotations were matched with the sequences from *Elaeis guineensis, Phoenix dactylifera, Musa acuminata* (Figure S3). According to GO annotation, most of the unigenes were assigned to biological processes (BP) (44%), cellular component (CC) (37%) and molecular function (MF) (19%) (Figure S4). In Figure S5A, there were 9,204 unigenes were divided into 25 specific categories by COG database. Similarly, 17,463 unigenes were assigned to 25 specific KOG categories (Figure S5B). The biochemical pathways were predicted by the KEGG database, a total of 6,334 (8.42%) unigenes were assigned into 118 pathways.

**Figure 1:**
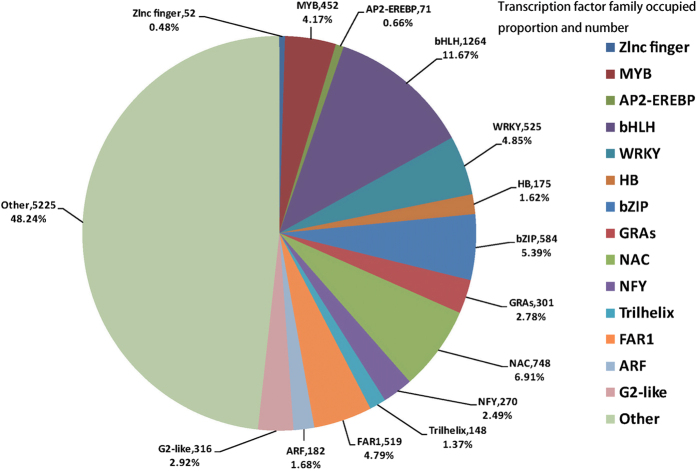
Transcription factor family occupied proportion and number in *Phormium tenax* transcriptome sequences.

Transcription factors are significant upstream regulatory proteins, which play an important role when plants are subjected to abiotic and biotic stress^8^. According to the prediction of transcription factors with PlantTFDB, a total of 10,832 transcription factor genes were obtained. The biggest family was bHLH (11.67%), followed by NAC (6.91%), and bZIP (5.39%), WRKY (4.85%), FAR1 (4.79%) (Fig. 1). It is worth mentioning that 5,225 (48.24%) genes belonged to others, mostly due to its lack of reporting in *Phormium tenax*.

**Figure 2:**
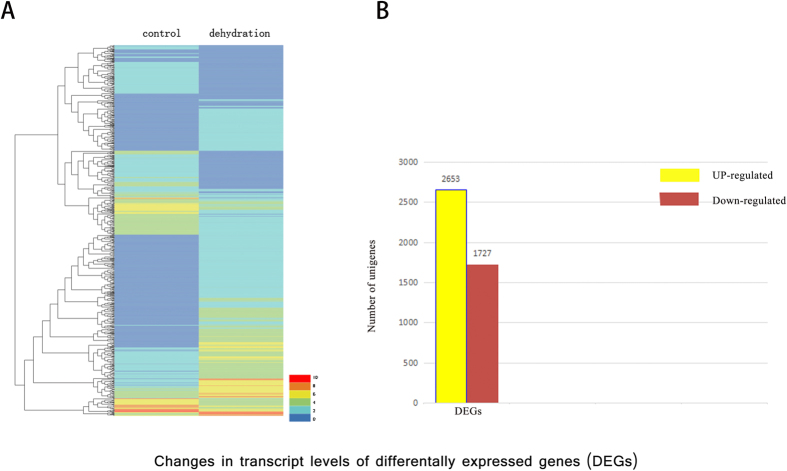
Summary graph of differential expression genes (DEGs). (**A**) Heat-maps of all DEGs. Columns and rows in heat maps represent samples and DEGs, respectively. (**B**) Number of up- and down-regulated DEGs.

The expression levels of 4,380 differentially expressed genes (DEGs) differed significantly under normal and drought conditions (Fig. 2A). Among them, 2,653 genes were up-regulated, while 1,727 were down-regulated (Fig. 2B). Totally, 2,698 genes were annotated finally under drought stress. Among them, the highest number were Nr (2,682) and Swiss-Prot (2,047), followed them were Pfam (1,816), GO (1,438), KOG (1,326), COG (819) and then KEGG (458). They are listed in Table S3.

Functional groups of gene related to drought response have been found with the parametric analysis of gene set enrichment. According to GO annotation with a total number of 1,438, we found that BP, CC and MF, respectively, accounted for 45.04%, 34.36% and 20.63% (Figure S6). In addition, we found ten significant nodes in BP and the most significant among them was “response to karrikin”. Other terms including “drug transmembrane transport”, “fatty acid biosynthetic process”, “glycolysis”, “response to salt stress” are listed in Table S4. In total, 819 DEGs were divided into 25 specific COG categories. The first three categories were “general function prediction only” (18.91%), “transcription” (9.46%) and “signal transduction mechanisms” (8.63%) (Figure S7). According to KEGG database, we found the highest number of DEGs was “plant-pathogen interaction” (8.38%), followed by “starch and sucrose metabolism” (7.78%), then “plant hormone signal transduction” (7.48%) and “ribosome” (7.19%) (Figure S8).

Some significant metabolic pathways enriched in clusters which related to drought stress were discovered. Abscisic acid (ABA) is a phytohormone that plays an important role in drought stress response of plants^9^. In this study, we observed two genes encoding protein phosphatase 2C (PP2C) [EC:3.1.3.16] were up-regulated during drought stress. The alpha-Linolenic acid (18:3) (α-LeA) metabolism pathway is important for Jasmine acid (JA) biosynthesis in higher plants^10^ and in this pathway, four genes were up-regulated in drought stress. Among genes associated with the metabolism of the JA, one gene encoding jasmonic acid-amino synthetase (JAR1) was down-regulated, while three genes encoding jasmonate ZIM domain-containing protein (JAZ) and one gene encoding v-myc myelocytomatosis viral oncogene homolog 2 (MYC2) were up-regulated in drought stress. The expression levels of gene encoding steroid 22-alpha-hydroxylase (CYP90B1) [EC:1.14.13.-] increased during drought stress, CYP90B1 is a key enzyme in the brassinosteroid biosynthesis^11^. In the starch and sucrose metabolism pathway, one gene encoding PYG [EC:2.4.1.1] was up-regulated in drought stress, while five genes encoding sacA [EC:3.2.1.26] were down-regulated. Besides, the changes of other enzymes were also very obvious. The expression levels of genes participate in phenylpropanoid biosynthesis decreased in drought stress, for example, four genes encoding cinnamyl-alcohol dehydrogenase (CAD) [EC:1.1.1.195] were down-regulated. Genes related to peroxidase [EC:1.11.1.7] and caffeoyl-CoA O-methyltransferase [EC:2.1.1.104] were both up-regulated in *Phormium tenax*. There were two genes encoding glutathione S-transferase (GST) [EC:2.5.1.18], one was up-regulated while the other was down-regulated in drought stress. Three genes encoding Gamma-glutamyl transpeptidase (GGT) [EC:2.3.2.2] were up-regulated in drought stress (Table S5).

**Figure 3:**
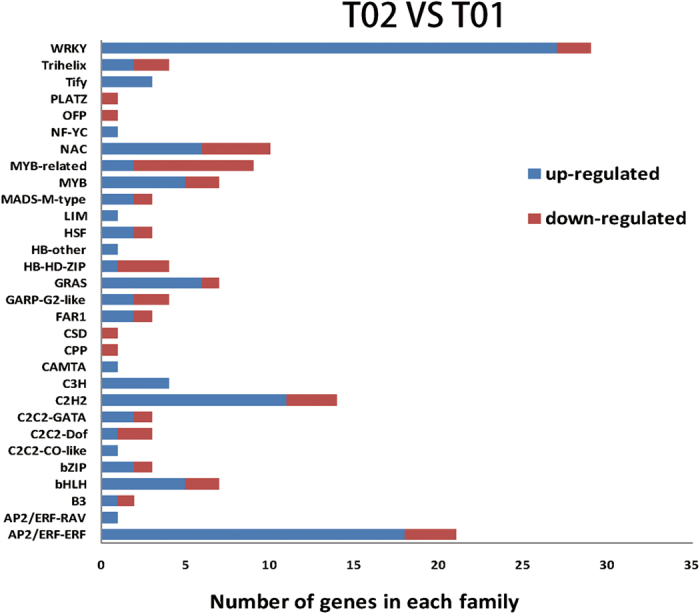
Transcription factors responsive to drought stress. Within each bar, number of up-regulated and down-regulated genes is shown in blue and red. T02 representative control group and T01 representative experimental group.

In this study, we found that transcription factors families were differentially expressed in response to drought treatment in *Phormium tenax*. The most abundant transcription factors families were WRKY (29), AP2/ERF-ERF (21), C2H2 (14), and NAC (10), followed by MYB-related (9), and MYB (7), bHLH (7), GRAS (7) (Fig. 3). The expression level of genes within each transcription factors family was inspected and different expression patterns were observed. Among them, 80 transcription factors genes were up-regulated and 24 were down-regulated in drought stress. Compared with known functional genes by establishing phylogenetic trees, several potential drought resistant genes belonging to the transcription factor families were discovered. There are three genes (Gene ID: c56991.graph_c0, c57387.graph_c0, c55976.graph_c0) belong to WRKY family (Figure S9A), four (Gene ID: c57500.graph_c0, c61891.graph_c0, c37958.graph_c0, c56737.graph_c0) belong to the MYB family (Figure S9B) and two (Gene ID: c58485.graph_c0, c58849.graph_c0) belong to the NAC family (Figure S9C). The result provided a reference to search for drought resistance genes in *Phormium tenax*.

**Figure 4:**
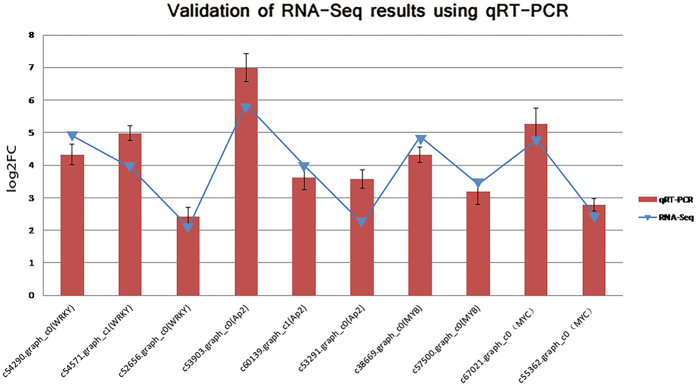
Validation of RNA-Seq results using qRT-PCR. The name of transcript factor behind each gene ID is determined by different expression gene function annotation.

To confirm the reliability of transcriptome sequence, ten DEGs were selected and analyzed using quantitative real-time PCR (qRT-PCR). The results showed almost the same level of fold changes between RNA-seq expression and qRT-PCR analyses (Fig. 4).

## Discussion

Plants have evolved regulatory mechanisms to adapt to arid environment in morphology and physiology. For example, in order to adapt to the arid environment, plants would decrease their leaf canopy to reduce water use or increase their root length to get access to deep water layers^1^. Osmotic adjustment substances in plants such as soluble proteins, soluble sugars and proline, antioxidative enzymes such as SOD, POD and CAT were both increased, which can improve the drought resistance of plants^12^. Therefore, the content of these osmotic adjustment substances and the activity of these enzymes can be used to estimate the characteristics of plants under drought conditions^12^. A conclusion can be drawn from the determination of the morphological indicators and physiological characteristics in this study: *Phormium tenax* has strong drought resistance.

Under drought stress, the result which whole transcriptome sequence analysis of DEGs showed that DEGs are related to drought stress in *Phormium tenax*. The results of GO enrichment in BP showed that the most significant among ten momentous nodes was “response to karrikin”. Karrikins are a fresh group of plant growth regulators discovered in the smoke of burning plant materials^13^. The seeds of “fire-ephemerals” can go through a series of cycles of imbibition and dehydration but remain dormant and karrikin-responsive^13^. Previous research has found that karrikins also increase stress tolerance of several crop species^14^. There are remarkable parallels in the signaling mechanisms of karrikins and other plant hormones, including auxins, jasmonates, and gibberellins^15^. A conclusion can be drawn that karrikins might play essential roles in *Phormium tenax* resistance to drought stress.

When plants are subject to drought stress, some genes are expressed in a large amount, and the related substances are produced to protect the plant through the drought period^12^. DEGs involved in the most important biochemical metabolic pathways and signal transduction pathways were identified by KEGG pathway. It is worth mentioning that “plant-pathogen interaction” represented the most significant terms, consistent with the previous researches which plant response to drought stress, such as *Macrotyloma uniflorum*^16^, *Reaumuria soongorica*^17^*, Arundo donax L*^18^. Previous studies indicated that once the plants were properly stimulated, such as drought, cold and wounding, they can be in an alarmed state and thus increase the defense against future pathogens^19^. In addition, it was also reported that paenibacillus polymyxa increases survival *Arabidopsis thaliana* under drought stress^10^. We found that several transcripts from JAZ, *WRKY25* and MYC2 members of the ‘Plant-pathogen interaction’ pathway were induced by drought stress in this study. We can speculate that there is a complex signaling network between drought stress and the activation of plant pathogen signal, which plays an important role in the tolerance of abiotic stress. Combined with the previous research^5^ and the results of KEGG enrichment in this study, the mechanism of drought resistance in *Phormium tenax* was explained from three aspects of metabolism and signaling of hormones, osmotic adjustment and reactive oxygen species metabolism.

### Metabolism and signaling of hormones in drought stress of *Phormium tenax*

Phytohormones are significant in the regulation of drought in *Phormium tenax*. Previous studies have indicated that phytohormones ABA is closely related to the regulation of plant drought resistance^20^. In this study, the PP2C associated with the ABA signaling pathway was up-regulated, suggesting that ABA responded positively to drought stress. In addition, the presence of ABA could be concurrent with their response to drought stress by mediating and channelizing many stress responsive genes that help the plants in their survival over stress^20^. This result also indicated that the response to drought stress in *Phormium tenax* is a conservative mechanism in the way of the metabolism of the ABA.

**Figure 5:**
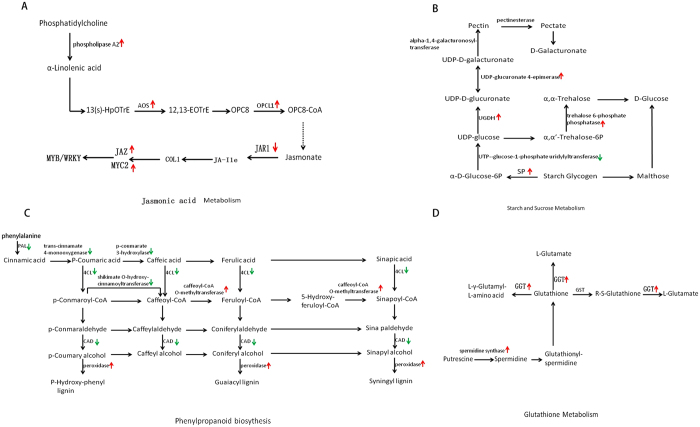
(**A**) Effect of drought stress on the expression of genes associated with JA metabolism. (**B**) Starch and sucrose metabolism. (**C**) Phenylpropanoid biosythesis. (**D**) Glutathione metabolism. Red arrow and green arrow indicate significantly up-regulated and down-regulated expression, respectively.

JA is affected by the drought stress. The fatty acid substrate of JA biosynthesis is α-LeA released from galactolipids of chloroplast membranes^21^. In rice, enhanced α-LeA metabolism is helpful to improve the drought resistance ability of drought-tolerant landraces/genotypes^22^. In this study, we discovered that the key enzyme, secretory phospholipase A2 (SPLA2 [EC:3.1.1.4]) was up-regulated under drought stress. Other two enzymes, both hydroperoxide dehydratase (AOS [EC:4.2.1.92]) and OPC-8:0 CoA ligase1 (OPCL1 [EC:6.2.1.-]) were also up-regulated. These results suggested that α-LeA metabolism pathway (Fig. 5A) was highly elevated by drought tolerance and promoted the release of α-LeA in the cell membrane, then transferred α-LeA into the oxylipin signaling molecule JA and activated numerous defensive genes. In addition, the JAR1 was down-regulated, suggesting that the drought stress induced the accumulation of JA. As a rapid chemical signal, JA can be transferred from shoot to root, and play a role in inducing gene expression. An obvious increase of JAZ under drought stress has also been found in this study, which might lead to activation of transcription factors such as WRKY and MYB. MYC2 is a significant regulator of various JA responses and mediates crosstalk with other pathways^23^. The MYC2 was up-regulated, suggesting that it has an important regulatory role in the metabolic process of JA.

Brassinosteroids (BRs) was widely involved in the growth and development of plants^11^. Besides, it is significant in varieties of plant adaptation and physiological processes to kinds of biotic and abiotic stresses^11^. Previous studies have shown that BRs could enhance tolerance to photo-oxidative and cold stresses in cucumber^24^. CYP90B1 [EC:1.14.13.-] is the key enzyme in the biosynthesis of bioactive BRs in plants^25^. In this study, CYP90B1 was up-regulated in *Phormium tenax* in drought stress. The result suggested that BRs biosynthesis in *Phormium tenax* played a positive role in the drought environment.

### Osmotic adjustment responding to dehydration

Osmotic adjustment substance is a non toxic small molecule organic compound, which is synthesized and accumulated in the cells to maintain the level of permeability *in vivo* under salt stress or drought stress^26^. Previous studies have shown that starch and sucrose metabolism plays an important role in osmotic adjustment^26^.

Starch is a non soluble sugar^26^. The key enzyme PYG [EC:2.4.1.1] related to the decomposition of starch was raised in drought conditions, on the contrary, the enzyme sacA [EC:3.2.1.26] related to the decomposition of sucrose was reduced. These results indicated that in drought stress, with the decomposition of non soluble sugar and the synthesis of soluble sugar, the intracellular solute could be accumulated and the ability of cells to absorb water could be enhanced, which will protect the *Phormium tenax* from hydropenia.

In this study, we found lots of up-regulated enzymes (Table S5) and some of them are encoded by more than one genes, such as alpha-1,4-galacturonosyltransferase (GalAT) [EC:2.4.1.43], pectinesterase [EC:3.1.1.11] and trehalose 6-phosphate phosphatase (TPP) [EC:3.1.3.12] (Fig. 5B). Pectin have multiple functions in plant growth, development and disease resistance including roles in cell–cell adhesion, wall porosity, cell elongation and wall extensibility^27^. GalAT and pectinesterase are the important enzymes in synthesis of pectin. The two enzymes were changing between up and down, under drought stress, which suggested that signal for regulation of pectin was very complex in *Phormium tenax.* TPP was a key enzyme which can strengthen drought tolerance in a variety of organisms^28^. In this study, two genes encoding TPP were both up-regulated, maybe shown that they play an important role in resisting drought stress, thus leading to changes in carbohydrate allocation and metabolism^29^. Other enzymes including UDPglucose 6-dehydrogenase [EC:1.1.1.22] and UDP-glucuronate 4-epimerase [EC:5.1.3.6] were both up-regulated. Under drought stress, the change in the up-regulated expression of genes related to starch and sucrose metabolism was amazing, suggesting that drought resistant invests more energy and resources into immediate defense needs.

In addition, amino acid compounds also play an important role in the osmotic adjustment. Previous studies have shown the great effect of plants drought tolerance on phenylpropanoid biosynthesis^11^. In this study, several enzymes were differentially expressed in phenylpropanoid biosynthesis under drought stress (Fig. 5C). Peroxidase [EC:1.11.1.7] and caffeoyl-CoA O-methyltransferase [EC:2.1.1.104] were both up-regulated, which indicated the positive relationship between drought stress and phenylpropanoid biosynthesis in *Phormium tenax*. But others like CAD [EC:1.1.1.195] was down-regulated in drought stress. Although there is little report about the function of CAD in drought stress, its function in plant development and defense against pathogens is great^30^. Four genes encoding CAD were all down regulated, which probably leading to reduced disease resistance in *Phormium tenax’s* drought stress. Phenylpropanoid biosynthesis has been confirmed to facilitate the synthesis of phenylpropanoid-based polymers, such as lignin and flavonoids^31^. The change of phenylpropanoid biosynthesis pathway in *Phormium tenax’s* drought stress may promote cell wall modification through affecting lignin biosynthesis, regulate anthocyanin biosynthesis by affecting anthocyanidins formation, then contribute to plant cuticle formation.

### Reactive oxygen species metabolism and drought stress

Under drought stress, the enzyme system of plant cells in the total change trend is synthetase activity decreased, proteolytic enzymes and some oxidoreductase activity increased^12^. Drought stress causes changes and disturbances of plant antioxidant enzyme system. Under drought conditions, the oxygen free radicals in plants increased. Many studies have indicated that one of the toxic effects of excess free radicals in plants is to cause membrane lipid peroxidation, which can damage the membrane system, and it will lead to the death of plant cells when the situation is serious^32^. Antioxidant enzymes such as SOD, POD, glutathione, etc., have the effect of eliminating free radicals and protecting the plant body. We found that four genes related to POD were up-regulated, which indicated that POD played an important role in drought stress (Table S6). In addition, these results suggested that genes related to glutathione metabolism were considerably changed during drought stress.

Previous studies have investigated the GST [EC:2.5.1.18] which existed in glutathione metabolism responses to abiotic stress and it is important in improving tolerance to salinity and drought stresses in rice and *Arabidopsis*^33^,^34^. In this study, GST was encoded by two genes, one was up-regulated and the other was down-regulated. In addition, the log_2_FC of up-regulated gene was 5.7 which investigated a close relationship between GST and drought tolerance in *Phormium tenax*. GGTs are the only enzymes known to hydrolyze the γ-glutamyl bond in glutathione^35^ which can hydrolyze the physiological antioxidant glutathione, indicating an involvement of the enzyme in the cellular defense mechanism against oxidative stress. In this study, genes encoding GST and GGT were up-regulation, showing that the two enzymes are at least partially favorable for improving drought resistance in *Phormium tenax* (Fig. 5D).

### Transcription factors responding to drought stress

Combined with the previous studies^36^, our result indicated that stress-related transcription factors, members of the WRKY, AP2/ERF-ERF, C2H2, NAC, MYB-related, MYB, bHLH and GRAS protein transcription factors have been well characterized for their roles in the regulation of drought tolerance. There were 27 up-regulated genes and 2 down-regulated genes in WRKY family. Previous studies have indicated that *AtWRKY33* regulated abiotic stress and that they may act as positive regulators in mediating the response of plants to ABA^37^, and four genes of *WRKY33* were discovered in this study. In addition, we found that the gene (gene ID: c57387.graph_c0) and *GmWRKY54* were similar, and *GmWRKY54* has been reported to be resistant to salt and drought resistance^38^. The plant-specific NAC (for NAM, ATAF1, 2 and CUC2) transcription factors have been implicated in diverse cellular processes involved in stress responses such as cold, salinity or drought as well as ABA signalling^39^. It was reported that *ANAC042* extends longevity and increases tolerance to heat stress in *Arabidopsis thaliana* when overexpressed^40^ and the gene (Gene ID: c58485.graph_c0) was found to have homology with *ANAC042* in this study. MYB also represents a high number of transcripts under drought, 7 genes were found to change under drought stress and they probably play the conserved or fundamental roles in *Phormium tenax* responses to stress. In this study, the gene (gene ID: c61891.graph_c0) and *CpMYB10* had high homology, it was reported that *CpMYB10* in *Arabidopsis* was mediating stress tolerance and altering ABA and Glc signaling responses^41^. GRAS transcription factors have a major function in plant growth and environmental adaptation, especially for the modulation of plant tolerance to stressorsα. For instance, *SCL7* has the characteristics of salt and drought tolerance in *Arabidopsis*^43^. In this study, *SCL23* is a member of GRAS transcription factors family, showed obvious up-regulation particularly in *Phormium tenax*. In addition, our study showed the number of up-regulated transcription factors was more than down-regulated transcription factors in drought stress, which indicated that the importance of transcription factors to drought stress in *Phormium tenax.*

## Materials and Methods

### Plant materials and stress treatment

Pot and artificial water control method were used to simulate the drought. *Phormium tenax*’s seedlings were obtained from Sichuan Agricultural University. The turfy earth and natural soil were mixed in proportion of 1:1. The mixture should be stacked 45 days after degassing. Then put the soil into the same size of plastic basins, 3 kg per pot, a total of 40 pots. The water content of the soil was controlled by 70% of the saturated water content of the soil (after the experiment, the saturated water content of the soil was 65.04%). One-year-old *Phormium tenax* which have consistent height and biomass were selected as materials and transplanted to plastic basins, 1 pot per pot. Watering according to the plant’s growth, the spilled water was poured back into the basin in order to maintain the soil moisture consistent. After 30 days of growth, 40 pot seedlings were randomly divided into two groups: control group (T02) and experimental group (T01). The initial soil moisture content of T02 remained unchanged, while stop watering the T01 until the soil moisture content was 25% of the soil saturated water content. The process took two weeks. Soil water content was measured every 2 days to replenish the amount of deficiency. Ten days after, *Phormium tenax* with different treatment were harvested for further use.

### Morphological indicators and physiological characteristics determination

Vernier caliper was used to measure the length of taproot, lateral root length and the shoot height. Leaves of T01 and T02 were harvested for determination of physiological characteristics. The activity of SOD was determined by Nitro-blue tetrazolium (NBT) photoreduction method^44^. The activity of POD was determined according to Li *et al*.^44^. CAT activity was measured by UV absorption method^44^. Soluble protein content was measured using Coomassie Brilliant Blue G-250 Dye Binding^44^. Soluble sugar content was determined according to anthrone colorimetric method^44^. Proline content was determined according to Bates *et al*.^44^. MDA content was measured as previously described^44^ with minor modifications.

### RNA extraction, library construction and sequencing

Each treatment (T01 and T02) was made up of three biological repeats. RNA was extracted from three biological repeats, respectively. And then the three individual RNAs were mixed in equal quantity to supply one pooled sample for the sequencing analysis. First of all, total RNA was extracted with Trizol Reagent (Invitrogen), following the manufacturer’s instructions. In order to get qualified samples for transcriptome sequencing, Nanodrop, Qubit 2.0 and Aglient 2100 were used to test the purity, concentration and integrity of the RNA. Messenger RNAs (mRNAs) were separated from the total RNA by Oligo (dT) and were cleaved into short fragments at random. Secondly, the first strand cDNAs were synthesized with random hexamer primers, the buffer, dNTPs, DNA polymerase I and RNase H were used to synthesize the second strand cDNAs. The cDNAs were purified with AMPure XP beads. End repair, add the A tail and connect the sequencing connector, fragment size selection on the purification of the double stranded cDNA to be carried out in turn. Lastly, the qualified cDNA libraries were constructed by PCR enrichment and sequenced by HiSeq2500 with sequencing read length of PE125. The raw sequencing data have been submitted to the NCBI Sequence Read Archive database with accession number SRP092290.

### Sequence analysis, *de novo* assembly and functional annotation

After filtering the raw data, high quality clean data was received for subsequent analysis. The unigene library of the *Phormium tenax* was obtained by sequence assembly of clean data. Trinity^45^ is an assembly software which specifically designed for high-throughput transcriptional sequencing. First, all clean reads whose Q30 (quality score) ≥92.59% were interrupted for short fragments (K-mer) by Trinity. Then, the K-mers were extended to long fragments (contig). Next, collection of fragments (component) was obtained by using the overlap between these contigs. Finally, using the method of De Bruijn map and sequencing Read information, we identified the transcript sequence in each component, respectively. The transcripts, their assembled sequences, expression value in each sample data have been submitted to the GEO database with accession number GSE92497 (The Platform number is GPL22804, the GSM2430721 represents drought condition T01 and the GSM2430722 represents normal condition T02).

In order to obtain the annotation information of unigene, the unigene sequences were compared against the Nr, Swiss-port, GO, COG, KOG, KEGG using BLAST firstly, and then compared with the Pfam database using HMMER.

### Differential expression analysis

In this study, EBSeq^46^ was used to analyze the differential expression, and the DEGs between the two samples were obtained. At the time of analysis, false discovery rate (FDR) was used as a key indicator for the screening of DEGs. In the screening process, FDR value which less than 0.01 and |log2 (fold change)| ≥ 2 as screening criteria. Fold change (FC), which represents the ratio of the expression of the two samples.

### Quantitative real-time PCR analysis

qRT-PCR was performed using SsoFast™ EvaGreen^®^ Supermix, following instruction of the manufacturer. Plant material was consistent with those used for RNA-seq. 10 DEGs belonging to the family of transcription factors were selected. Their primers are listed in Table S7. qRT-PCR was performed with three biological replicates. The expression pattern of DEGs was analyzed by melting furnace curve.

## Conclusions

The high-throughput sequencing technology can fully obtain almost all the transcript sequence information of a species specific tissues or organs in a certain state, has been widely used in basic research, clinical diagnosis and drug development. In this study, transcriptome sequencing analysis of *Phormium tenax* was performed through RNA-seq technology. Besides, we explained drought-resistant mechanism of *Phormium tenax* from the molecular level. These results are useful for the study of the mechanism of drought resistance in plants and provide excellent genetic resources for drought-resistant crops.

## Additional Information

**How to cite this article:** Bai, Z.-y. *et al*. Whole-transcriptome sequence analysis of differentially expressed genes in *Phormium tenax* under drought stress. *Sci. Rep.* **7**, 41700; doi: 10.1038/srep41700 (2017).

**Publisher's note:** Springer Nature remains neutral with regard to jurisdictional claims in published maps and institutional affiliations.

## Supplementary Material

Supplementary Dataset 1
