# Supplementary material for: Whole-transcriptome sequence analysis of differentially expressed genes in Phormium tenax under drought stress
Source: Sci Rep. 2017 Jan 30;7:41700. doi: 10.1038/srep41700 (PMC5278365; doi:10.1038/srep41700)
Supplement: Supplementary Dataset 1 [file srep41700-s1.doc]

Whole-transcriptome sequence analysis of differentially expressed genes in *Phormium tenax* under drought stress

Short title：Transcriptome sequence analysis of *Phormium tenax* under drought stress

Zhen-yu Bai, Tong Wang, Yin-huan Wu, Ke Wang, Qian-yu Liang, Yuan-zhi Pan, Bei-bei Jiang, Lei Zhang, Guang-li Liu, Yin Jia, Qing-lin Liu*.

Department of Ornamental Horticulture, Sichuan Agricultural University, 211 Huimin Road, Wenjiang District, Chengdu, Sichuan 611130, P.R. China.

*Corresponding author; E-mail: qinglinliu@126.com;

Tel/Fax: +86−28−86290881.

**Supplementary Table S1** Summary of Illumina transcriptome assembly for *Phormium tenax.*

| Length Range | Contig | Transcript | Unigene |
| --- | --- | --- | --- |
| 200-300 | 4,018,976(98.91%) | 50,591(28.80%) | 37,367(49.65%) |
| 300-500 | 19,826(0.49%) | 32,592(18.56%) | 16,034(21.30%) |
| 500-1000 | 12,610(0.31%) | 38,439(21.88%) | 10,806(14.36%) |
| 1000-2000 | 8,262(0.20%) | 37,949(21.61%) | 7,776(10.33%) |
| 2000+ | 3,745(0.09% | 16,078(9.15%) | 3,282(4.36%) |
| Total Number | 4,063,419 | 175,649 | 75,265 |
| Total Length | 223,179,195 | 150,366,644 | 42,590,937 |
| N50 Length | 49 | 1,396 | 919 |
| Mean Length | 54.92 | 856.06 | 565.88 |

**Supplementary Table S2** Functional annotation of *Phormium tenax* transcriptome.

| Annotated databases | Unigene | ≥300nt | ≥1000nt |
| --- | --- | --- | --- |
| COG | 9,204 | 7,401 | 4,556 |
| GO | 16,809 | 13,039 | 6,455 |
| KEGG | 6,334 | 5,099 | 2,756 |
| KOG | 17,463 | 13,615 | 6,757 |
| Pfam | 18,459 | 15,472 | 8,677 |
| Swiss-Prot | 20,891 | 16,730 | 8,127 |
| nr | 30,027 | 22,962 | 10,311 |
| All | 30,814 | 23,098 | 10,325 |

**Supplementary Table S3** Differential expression genes annotation of *Phormium tenax* transcriptome

| Annotated | COG | GO | KEGG | KOG | Pfam | Swiss-prot | nr |
| --- | --- | --- | --- | --- | --- | --- | --- |
| 2,698 | 819 | 1,438 | 458 | 1,326 | 1,816 | 2,047 | 2,682 |

**Supplementary Table S4** GO enrichment biochemistry top10

| GO ID | Term | Annotated  genes | Significant  genes | Expected | KS |
| --- | --- | --- | --- | --- | --- |
| 0080167 | response to karrikin | 66 | 15 | 5.65 | 1.60E-06 |
| 0006855 | Drug transmembrane transport | 63 | 8 | 5.4 | 1.40E-05 |
| 0006633 | Fatty acid biosynthetic process | 194 | 35 | 16.62 | 0.00042 |
| 0006096 | glycolysis | 175 | 17 | 14.99 | 0.00047 |
| 0009651 | response to salt stress | 364 | 33 | 31.18 | 0.0005 |
| 0030261 | chromosome condensation | 19 | 0 | 1.63 | 0.00067 |
| 0009853 | photorespiration | 86 | 2 | 7.37 | 0.00085 |
| 0055114 | oxidation-reduction process | 1444 | 150 | 123.71 | 0.00118 |
| 0048731 | system development | 1228 | 78 | 105.2 | 0.00119 |
| 0015698 | inorganic anion transport | 84 | 22 | 7.2 | 0.00146 |

**Supplementary Table S5** Differentially expressed genes classified by KEGG pathway

| KEGG  pathway | KEGG  annotation | Gene ID | Log_2_FC | KO |
| --- | --- | --- | --- | --- |
| Ko04075  Plant hormone signal transduction | PP2C [EC:3.1.3.16];  JAR1;  JAZ;  MYC2; | c39046.graph_c0;  c59144.graph_c0;  c44105.graph_c0;  c48623.graph_c0;  c51091.graph_c0;  c65972.graph_c1;  c55362.graph_c0; | 2.17;  2.39;  -2.31;  3.82;  4.43;  6.17;  2.47; | K14494  K14506  K13464  K13422 |
| ko00592  alpha-Linolenic acid  metabolism | phospholipase A2 (SPLA2)[EC:3.1.1.4];  hydroperoxide dehydratase (AOS)[EC:4.2.1.92];  OPC-8:0 CoA ligase 1 (OPCL1)[EC:6.2.1.-]; | c64401.graph_c0;  c61296.graph_c0;  c49155.graph_c0;  c49155.graph_c1; | 2.03;  2.89;  3.04;  3.24; | K14674  K01723  K10526 |
| ko00905  Brassinosteroid biosynthesis | Steroid 22-alpha-hydroxylase  (CYP90B1, DWF4) [EC:1.14.13.-]; | c52144.graph_c0; | 4.00; | K09587 |
| ko00500  Starch and sucrose  metabolism | UDPglucose  6-dehydrogenase [EC:1.1.1.22];  UDP-glucuronate 4-epimerase [EC:5.1.3.6];  alpha-1,4-galacturonosyltransferase [EC:2.4.1.43];  pectinesterase [EC:3.1.1.11];  trehalose 6-phosphate phosphatase (TPP)[EC:3.1.3.12];  starch phosphorylase (PYG)[EC:2.4.1.1];  SacA [EC:3.2.1.26.]; | c63742.graph_c1;  c55843.graph_c0;  c36159.graph_c0;  c55587.graph_c0;  c63655.graph_c1; c37104.graph_c0;  c54495.graph_c0;  c58677.graph_c0;  c59333.graph_c0; c29288.graph_c0;  c54392.graph_c1;  c64847.graph_c0;  c58481.graph_c0;  c14512.graph_c0;  c48906.graph_c0;  c51361.graph_c0;  c51827.graph_c0;  c61021.graph_c0 | 2.05;  4.96  2.49;  2.09;  2.04; -5.08;  8.45;  3.50;  6.12;  -3.39;  4.84;  3.63;  2.21;  -4.36;  -2.59;  -2.15;  -2.22;  -3.92; | K00012  K08679  K13648  K01051  K01087  K00688  K01193 |
| ko00940  Phenylpropanoid  biosythesis | cinnamyl-alcohol dehydrogenase (CAD)[EC:1.1.1.195];  peroxidase [EC:1.11.1.7];  caffeoyl-CoA O-methyltransferase [EC:2.1.1.104]; | c30240.graph_c1;  c52319.graph_c0;  c59765.graph_c0;  c59765.graph_c1;  c28269.graph_c0;  c57806.graph_c0;  c38538.graph_c0;  c54553.graph_c0; | -2.52;  -2.75;  -2.57;  -2.46;  2.91;  3.52;  -3.98;  2.19; | K00083  K00430  K00588 |
| ko00480  Glutathione metabolism | gamma-glutamyltranspeptidase / glutathione hydrolase (GGT)[EC:2.3.2.2];  glutathione S-transferase (GST)[EC:2.5.1.18]; | c54014.graph_c0;  c66134.graph_c0;  c66148.graph_c0;  c66489.graph_c0; c58202.graph_c0; | 2.04;  8.87;  7.01;  5.78;  -2.86; | K00681  K00799 |

**Supplementary Table S6** Four up-regulated genes associated with POD

| Gene ID | FDR | log2FC | regulated |
| --- | --- | --- | --- |
| c28269.graph_c0 | 0.00000138 | 2.915205594 | up |
| c57806.graph_c0 | 0.0000000063 | 3.522002448 | up |
| c57081.graph_c1 | 0.0000208 | 2.819467912 | up |
| c63278.graph_c2 | 0.00000000381 | 3.98896753 | up |

**Supplementary Table S7** The primers of 10 DEGs for qRT-PCR

| Gene ID | Transcription factors family | Primers(5’-3’) |
| --- | --- | --- |
| c54290.graph_c0 | WRKY | Forward: TCCTCTGTACTCCTACTGTGAC  Reverse: TCCAGCCCTGTAAAATCTTCC |
| c54571.graph_c1 | WRKY | Forward: CGTCACCTCTGATTATGGCTC  Reverse: CCACCTTTATCCTCTTCAGCTC |
| c52656.graph_c0 | WRKY | Forward: GCTGATCATAACCTACACCGG  Reverse: CTACTCCCTTCCGTTTTCTCC |
| c53903.graph_c0 | AP2 | Forward: CATTCCCACCCTACTCCTTC  Reverse: TTCCTCTTGATTGGCACCG |
| c60139.graph_c1 | AP2 | Forward:ACCTAAAGAACATTTTCAGCACATG  Reverse: GCATGTAGAAGACTTTTCCGGG |
| c53291.graph_c0 | AP2 | Forward: CAAGGCCAAGACGAACTTTG  Reverse: GAGGTCCGAGAAACAGATCC |
| c38669.graph_c0 | MYB | Forward: GCCCTACATGTATCAAGCTAGC  Reverse: CATCCAAGTTCCATAGACTCCC |
| c57500.graph_c0 | MYB | Forward: TTGAGGTGTGGGAAGAGTTG  Reverse: TTCTCTGGTGAAGTTGCCTC |
| c67021.graph_c0 | MYC | Forward: GAGAAAATCAACGCCATCCTG  Reverse: CCTTCAGATCGCATCCTTCAAG |
| c55362.graph_c0 | MYC | Forward: CAATTTCTGCGATAACCAGCG  Reverse: TTCGTCTTTGCTTCTCACTCTC |


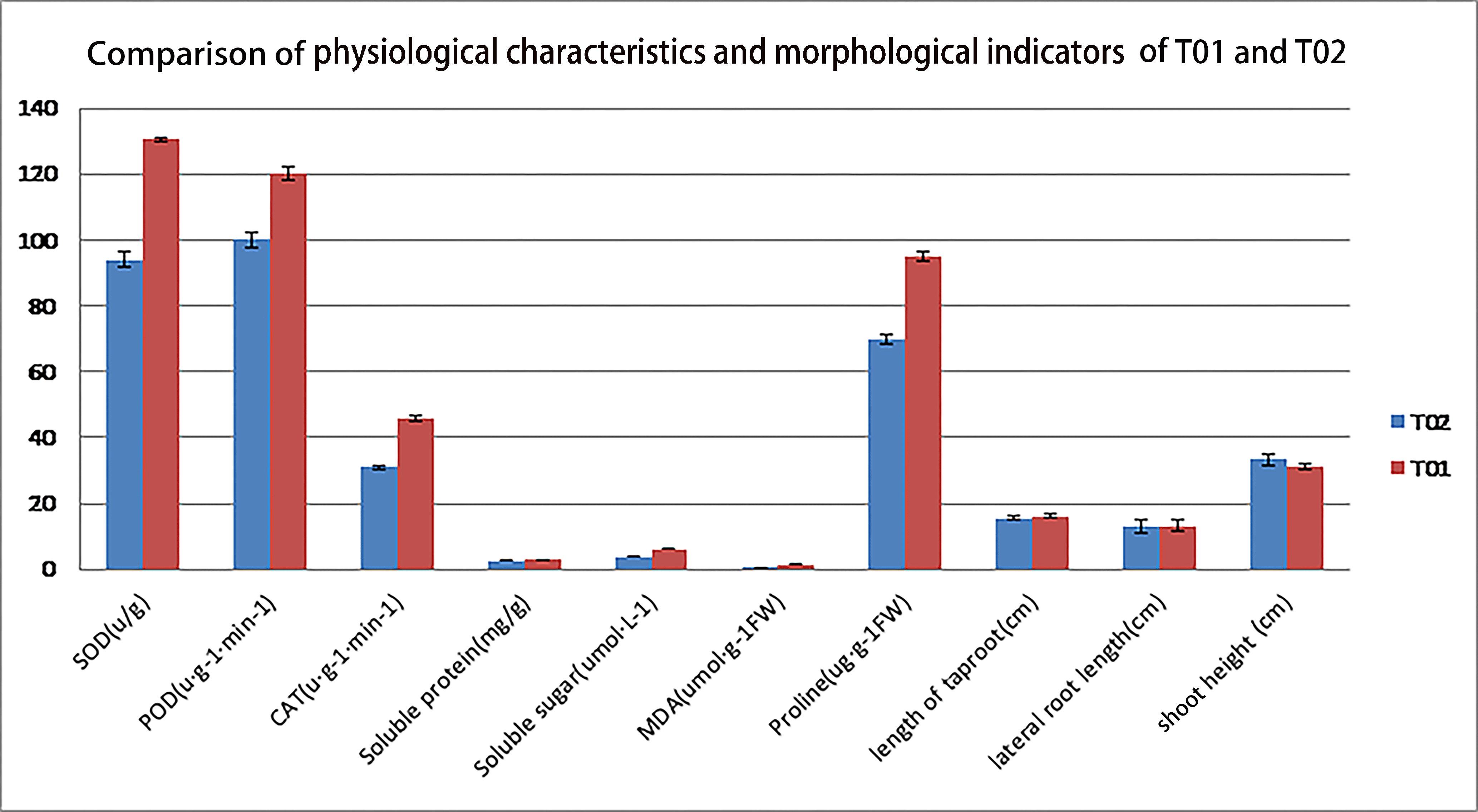


**Supplementary Figure S1** Comparison of physiological characteristics and morphological indicators of T01 and T02.


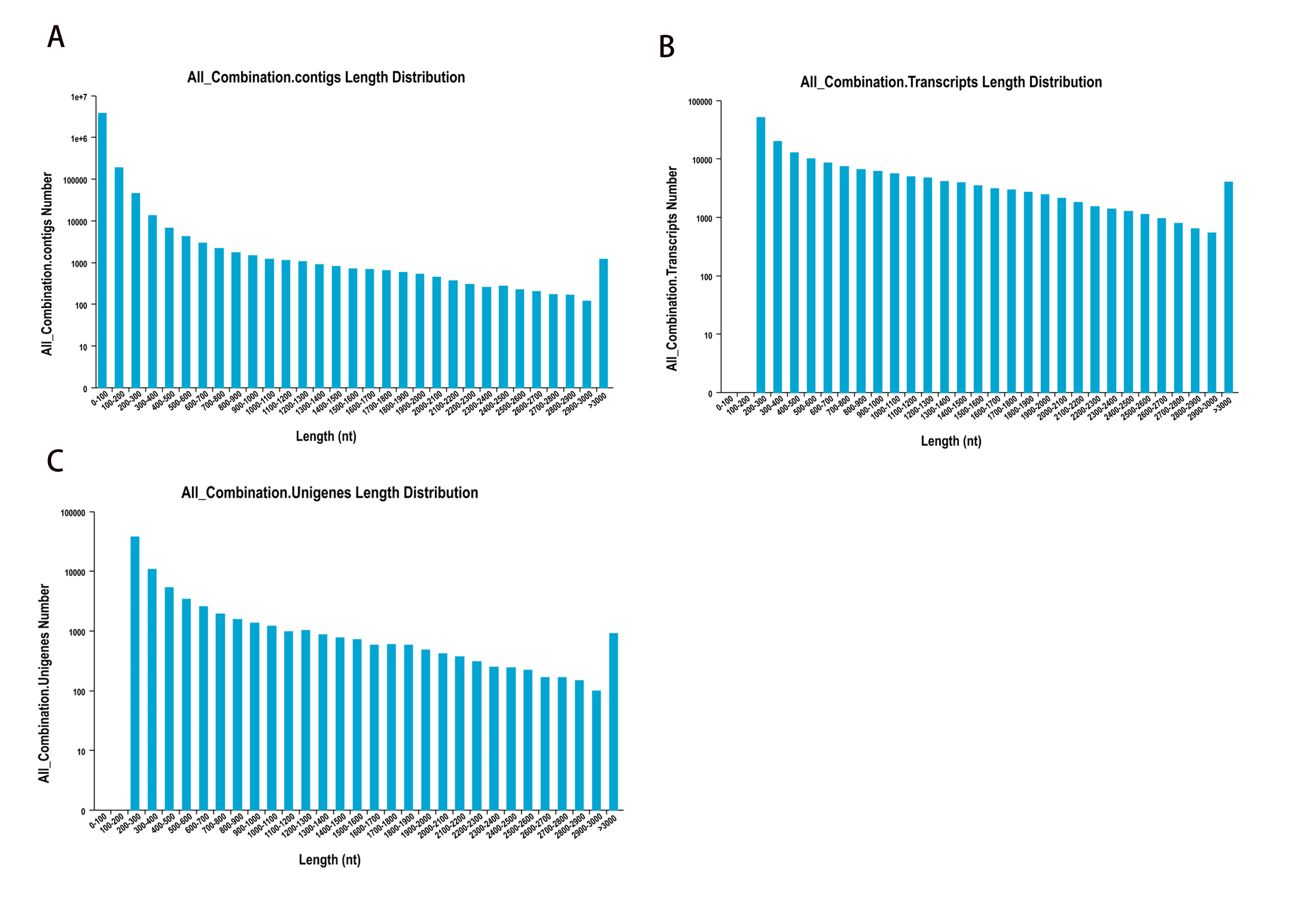


**Supplementary Figure S2** Overview of the Phormium tenax transcriptome sequencing and assembly. (A) Length distribution of Phormium tenax contigs. (B) Length distribution of Phormium tenax transcripts. (C) Size distribution of Phormium tenax unigenes.


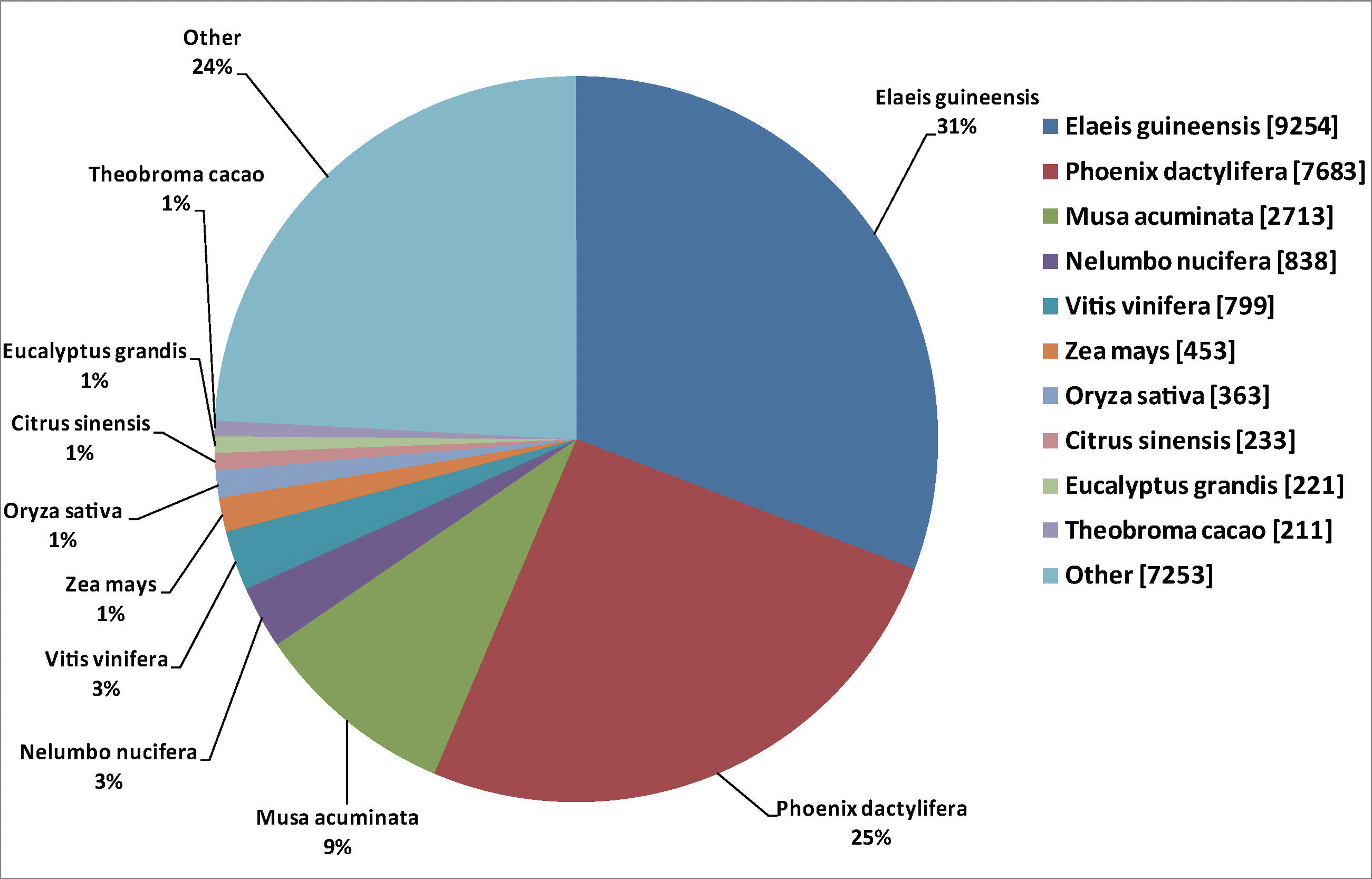


**Supplementary Figure S3** Species distribution of *Phormium tenax* from the velvet *de novo* assembly.


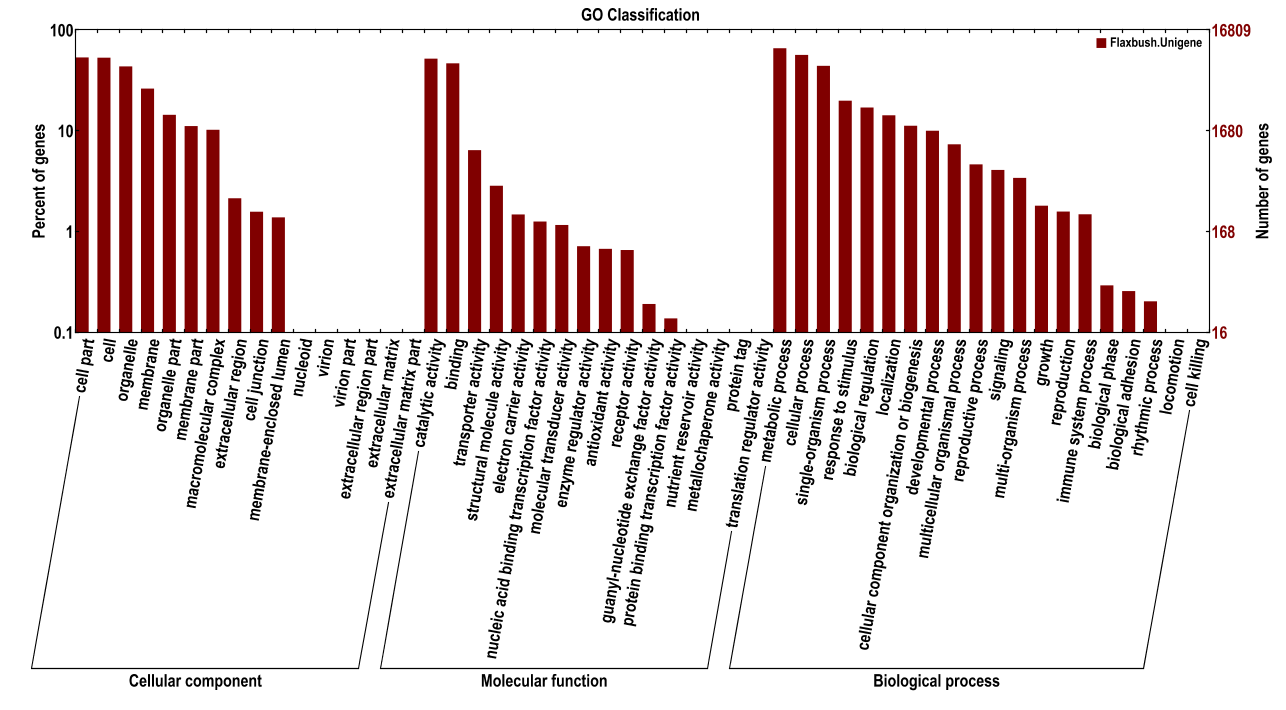


**Supplementary Figure S4** Functional annotation of assembled sequences based on GO categrization. GO analysis was performed at the level two for three main categories (cellular component, molecular function and biological process).


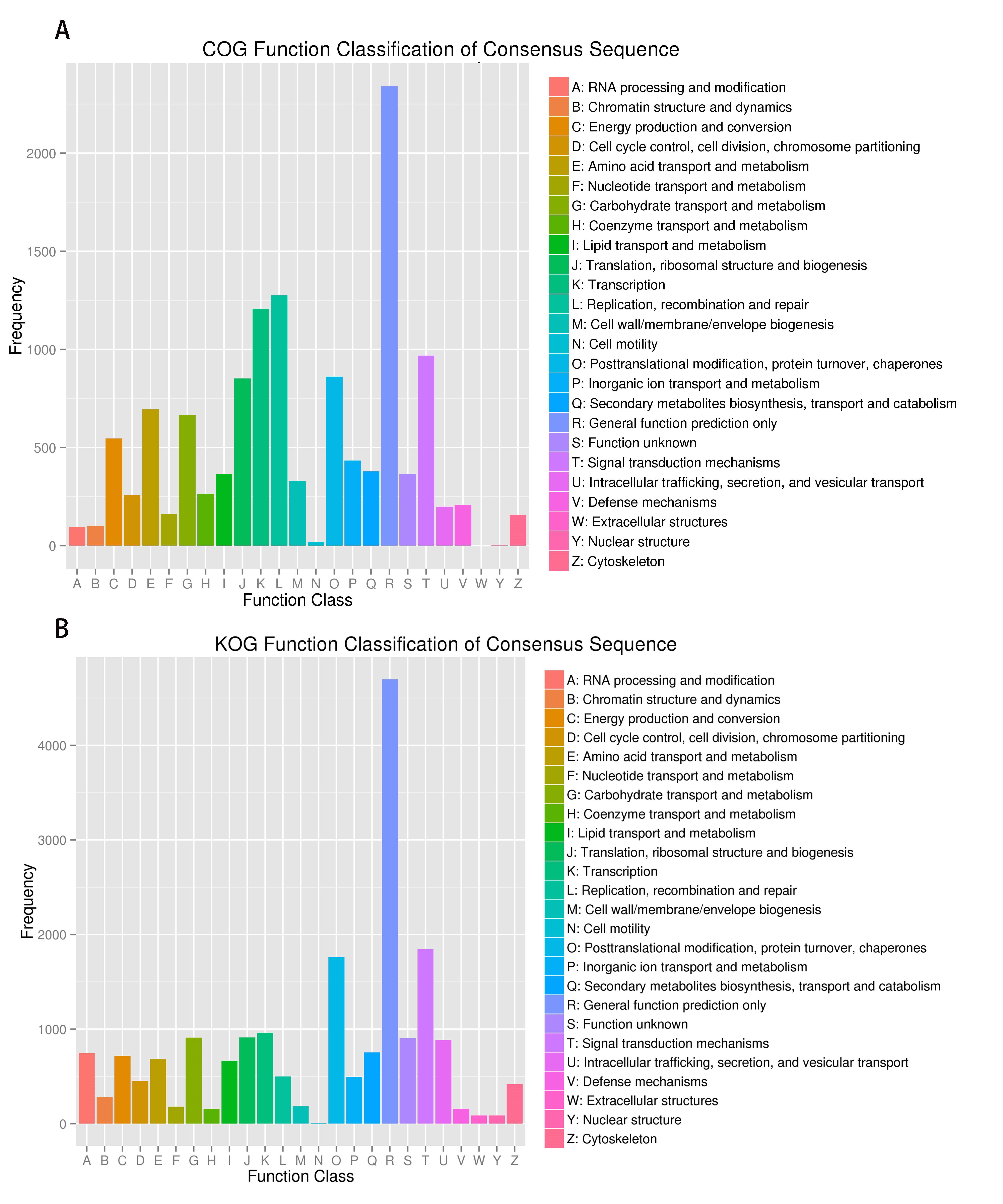


**Supplementary Figure S5** (A) Clusters of COG classification. (B) Clusters of KOG classification.


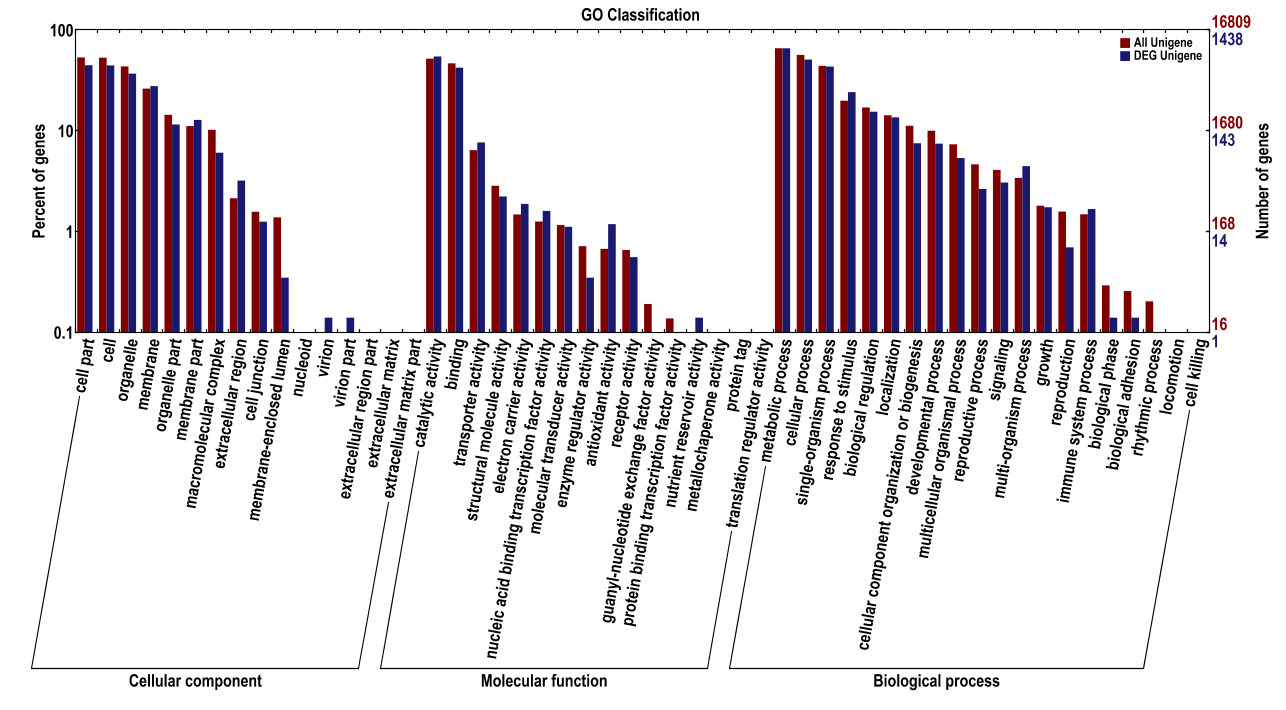


**Supplementary Figure S6** Fig Annotation of DEGs: Functional annotation of assembled sequences based on GO categorization. GO analysis was performed at the level two for three main categories (cellular component, molecular function and biological process).


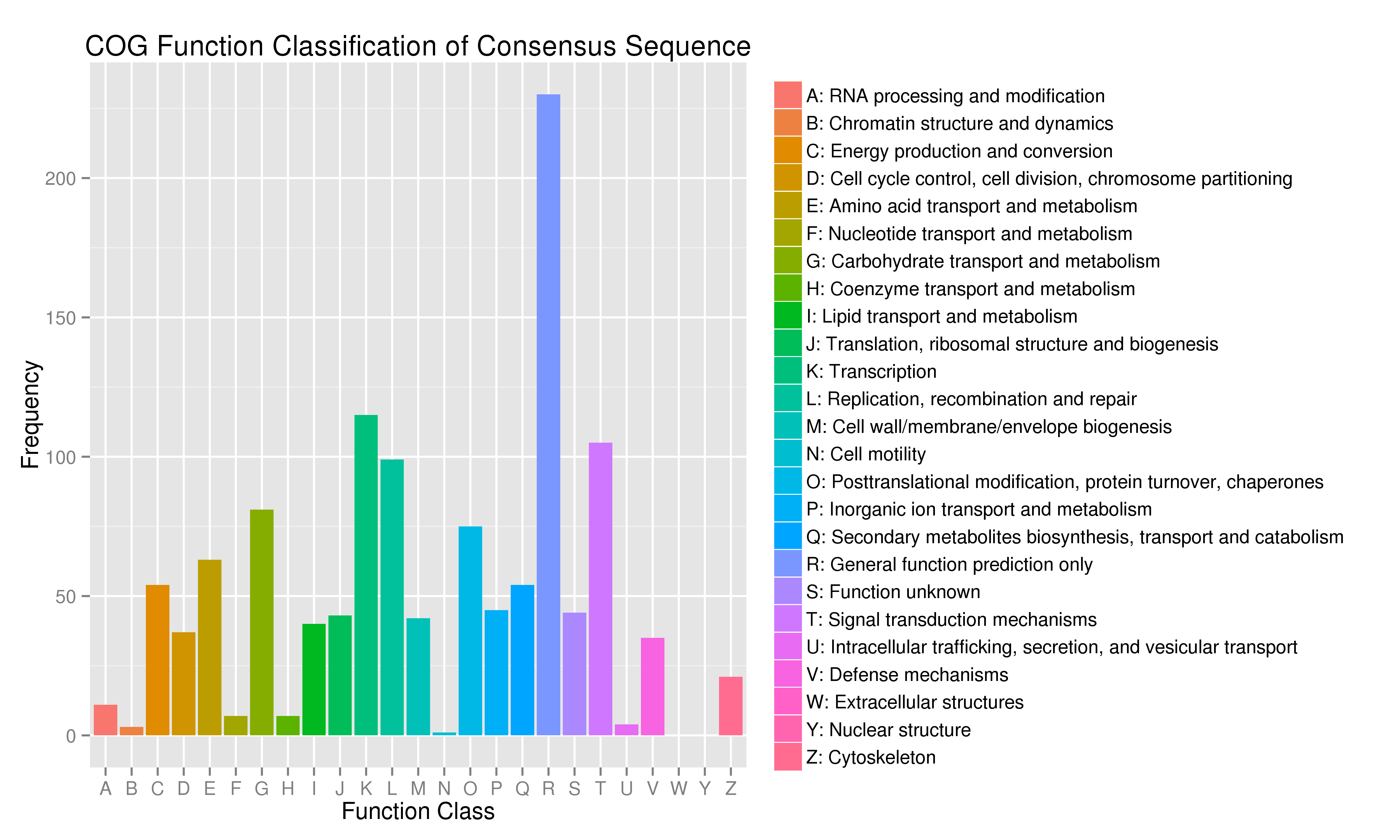


**Supplementary Figure S7** Annotation of DEGs: Clusters of COG classification.


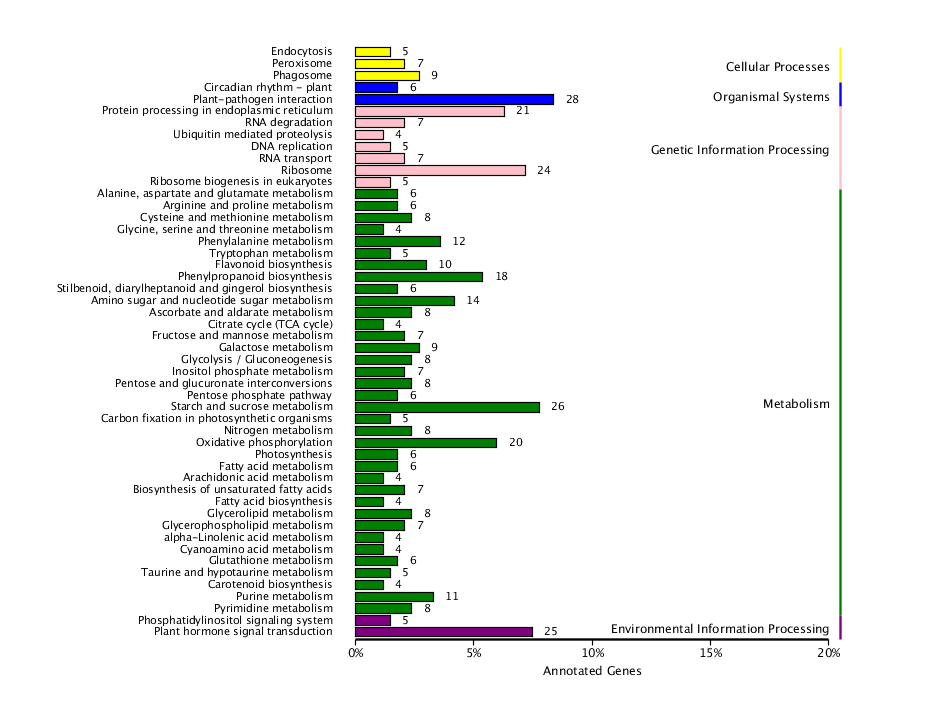


**Supplementary Figure S8** Annotation of DEGs: Clusters of KEGG classification.


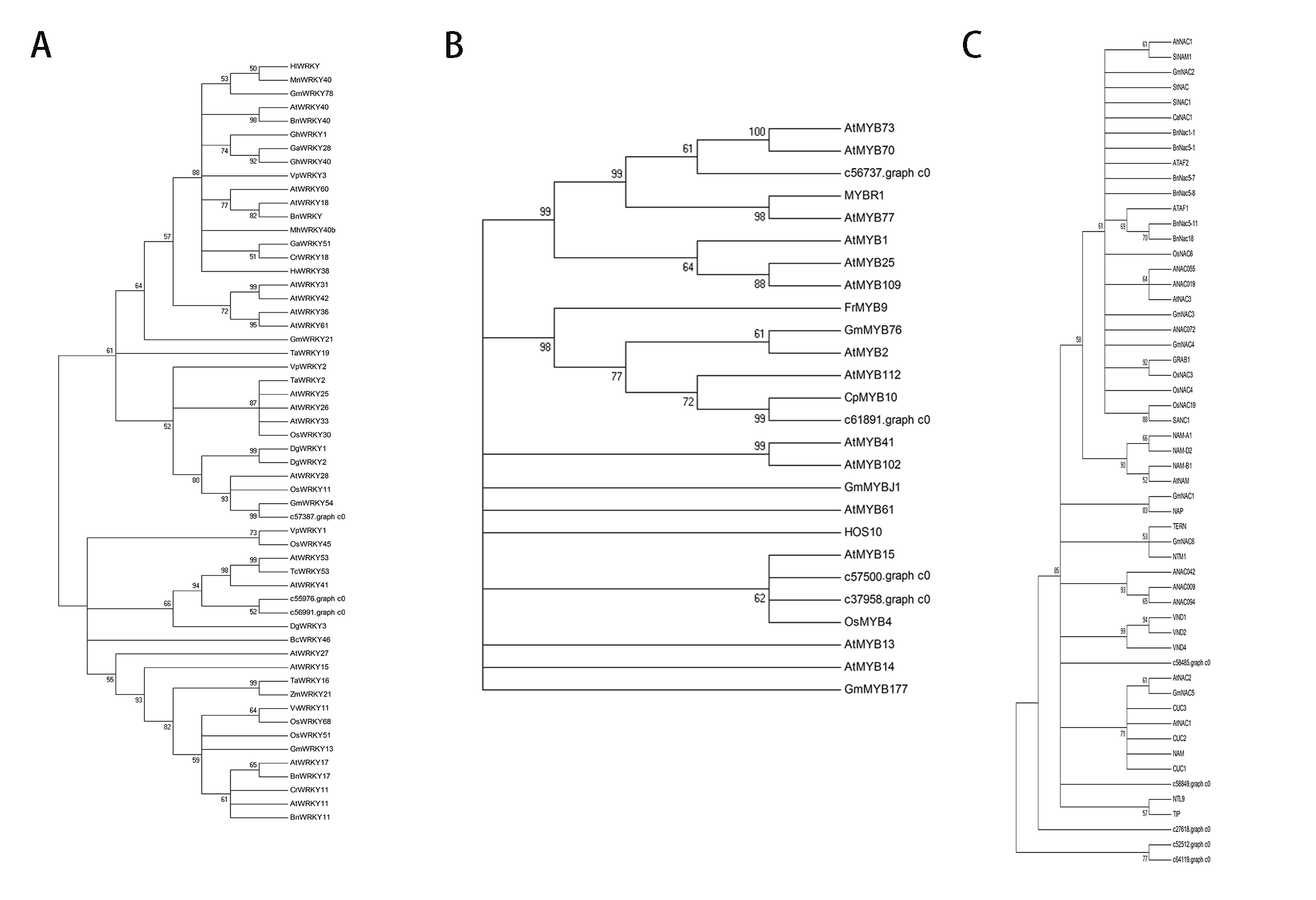


**Supplementary Figure S9** Potential drought resistance gene in *Phormium tenax*: (A) Comparison of known functional genes belonging to WRKY family. (B) Comparison of known functional genes belonging to MYB family. (C) Comparison of known functional genes belonging to NAC family.
